# Synthesis of Nanoparticles by Spark Discharge as a Facile and Versatile Technique of Preparing Highly Conductive Pt Nano-Ink for Printed Electronics

**DOI:** 10.3390/nano11010234

**Published:** 2021-01-18

**Authors:** Alexey A. Efimov, Pavel V. Arsenov, Vladislav I. Borisov, Arseny I. Buchnev, Anna A. Lizunova, Denis V. Kornyushin, Sergey S. Tikhonov, Andrey G. Musaev, Maxim N. Urazov, Mikhail I. Shcherbakov, Denis V. Spirin, Victor V. Ivanov

**Affiliations:** 1 Moscow Institute of Physics and Technology, National Research University, 141701 Dolgoprudny, Russia; arsenov@phystech.edu (P.V.A.); borisov.vi@mipt.ru (V.I.B.); buchnev.ai@mipt.ru (A.I.B.); lizunova.aa@mipt.ru (A.A.L.); korniushin.d@mipt.ru (D.V.K.); sergei.s.tikhonov@phystech.edu (S.S.T.); kuzemin@phystech.edu (A.G.M.); urazov.mn@mipt.ru (M.N.U.); ivanov.vv@mipt.ru (V.V.I.); 2 Kotelnikov Institute of Radioengineering and Electronics of Russian Academy of Sciences, 125009 Moscow, Russia; info@irtis.ru; 3 IRTIS Ltd., 105120 Moscow, Russia; den@irtis.ru

**Keywords:** spark discharge, platinum nanoparticles, Pt nano-ink, aerosol jet printing, resistivity

## Abstract

A cost-effective, scalable and versatile method of preparing nano-ink without hazardous chemical precursors is a prerequisite for widespread adoption of printed electronics. Precursor-free synthesis by spark discharge is promising for this purpose. The synthesis of platinum nanoparticles (PtNPs) using a spark discharge under Ar, N_2_, and air has been investigated to prepare highly conductive nano-ink. The size, chemical composition, and mass production rate of PtNPs significantly depended on the carrier gas. Pure metallic PtNPs with sizes of 5.5 ± 1.8 and 7.1 ± 2.4 nm were formed under Ar and N_2_, respectively. PtNPs with sizes of 18.2 ± 9.0 nm produced using air consisted of amorphous oxide PtO and metallic Pt. The mass production rates of PtNPs were 53 ± 6, 366 ± 59, and 490 ± 36 mg/h using a spark discharge under Ar, N_2_, and air, respectively. It was found that the energy dissipated in the spark gap is not a significant parameter that determines the mass production rate. Stable Pt nano-ink (25 wt.%) was prepared only on the basis of PtNPs synthesized under air. Narrow (about 30 μm) and conductive Pt lines were formed by the aerosol jet printing with prepared nano-ink. The resistivity of the Pt lines sintered at 750 °C was (1.2 ± 0.1)·10^−7^ Ω·m, which is about 1.1 times higher than that of bulk Pt.

## 1. Introduction

Currently, methods of manufacturing electronic circuits using printed technologies are actively developing for applications in antennas [1,2], transistors [3,4,5], sensors [6,7,8], displays [9,10], solar cells [11,12,13] and others. Printing technologies are based on the selective deposition of material in the form of nano-ink onto a substrate using printing equipment such as inkjet [14,15,16], aerosol jet [17,18,19,20], screen [21,22] and other printers. The use of printing technologies provides a significant reduction in the cost and time of manufacturing electronic devices compared to traditional processes in electronics such as lithography, etching and sputtering [23,24]. Moreover, printing technologies open up new possibilities for the production of flexible and lightweight electronic devices on substrates of polymers, paper or textiles [25,26,27,28,29]. Obviously, further progress in printing technology will depend on the state of development in printing methods, substrate and nano-ink properties.

Nowadays, chemical reduction is the most studied method for producing nano-ink for printing technologies [30,31]. This method is effective for preparing nano-ink with monodisperse particles of the required size and shape under controlled conditions [32,33]. On the other hand, this method is a long multi-stage process with a large amount of chemical waste and it requires the use of toxic and expensive precursors [33,34,35]. Moreover, the nanoparticles produced by this method have surface contaminants that reduce the conductivity of the nano-ink [36,37]. Due to these disadvantages, there is a challenge to develop an environmentally friendly method for producing nano-ink without the use of toxic and expensive precursors.

It is known that the synthesis of nanoparticles using a spark discharge does not require the use of precursors, in contrast to the method of chemical reduction [38,39,40]. Spark discharge synthesis is a simple and versatile method for producing nanoparticles, since it requires only gas, electrodes, and electricity. Moreover, spark discharge synthesis is carried out in a pure gas atmosphere that protects the nanoparticles from contamination [39,41]. In this regard, the spark discharge synthesis can become an eco-friendly and simple method of producing nanoparticles for nano-inks. Despite numerous studies of the method for the synthesis of nanoparticles by spark discharge, not a single study is known for the use of this method for the preparation of nano-ink. Thus, this work for the first time proposes using a spark discharge as a source of chemically pure nanoparticles for the preparation of conducting nano-ink.

We have chosen platinum as a material for preparing nano-ink. Platinum is a noble metal with excellent catalytic properties and high chemical stability [42,43]. In printed technologies, platinum is promising for the manufacture of corrosion-resistant current-carrying contacts [44,45], elements of gas [46,47], temperature [48,49] and biological [50,51] sensors. In our experiments, platinum nanoparticles (PtNPs) were synthesized by spark generator during electrical erosion of platinum electrodes in an atmosphere of Ar, N_2_, and air. The collection of nanoparticles for the preparation of Pt nano-ink was carried out on a fibrous filter, followed by cleaning and dispersing the nanoparticles into a liquid. The real-time measurement of the particle size distribution was performed using an aerosol spectrometer. The size, morphology, and elemental composition of the synthesized nanoparticles were studied using transmission and scanning electron microscopes and an X-ray photoelectron spectrometer. The prepared Pt nano-ink was tested and applied to form the conductive lines using an aerosol jet printer. The profile and resistivity of the formed lines were determined using an optical profilometer, a current source and a multimeter, respectively.

## 2. Materials and Methods

### 2.1. Synthesis of PtNPs by Spark Discharge

**Figure 1:**
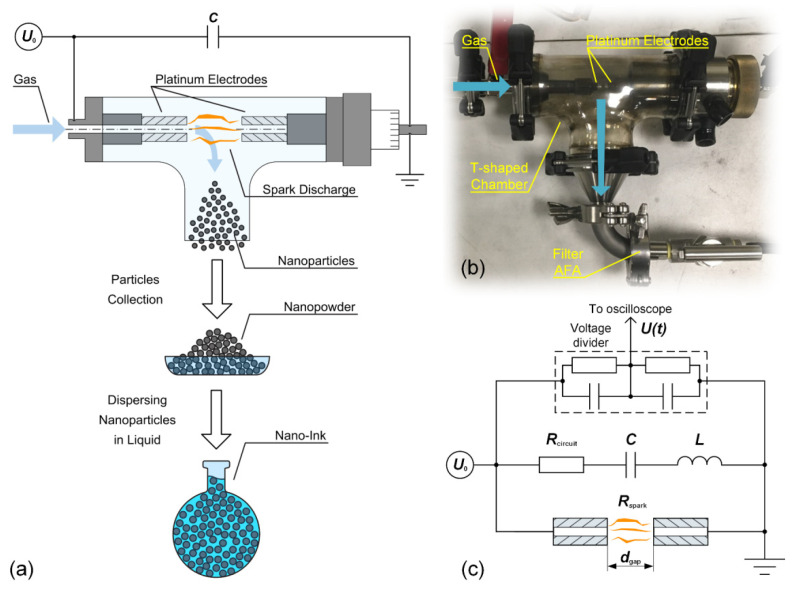
(**a**) Scheme of gas-phase synthesis of nanoparticles for the preparation of platinum nano-ink, (**b**) photograph and (**c**) equivalent electrical circuit of the spark discharge generator (SDG).

In this work, we investigated the preparation of nano-ink based on PtNPs synthesized by a spark discharge generator (SDG) in the atmosphere of three gases—Ar, N_2_ and air. Figure 1a,b show a scheme of the preparation of nano-ink and the photograph of an SDG.

The SDG consisted of a T-shaped discharge chamber with a volume of 400 cm^3^ and two holders for electrodes. The carrier gas entered the discharge chamber through the coaxial channel of one of the electrode holders (Figure 1a,b). This gas supply configuration ensured efficient removal of nanoparticles from the discharge zone. The second electrode holder was equipped with a micrometer screw to control the gap distance between the electrodes with a linear step of 0.05 mm. Hollow electrodes in the form of cylinders with a mass content of platinum of about 99.97% were used as a starting material for the preparation of nanoparticles. The length and outer and inner diameters of the electrodes were 20, 5.5, and 3.7 mm, respectively. The discharge chamber, tubes, and electrodes holders were made of heat-resistant glass, stainless steel, and brass to ensure the gas path’s high chemical purity. The carrier gas was supplied to the discharge chamber at a pressure of 2 bars and a flow rate of 3.5 L/min, which was set using a flow meter (PFM2-SP, SMC Inc., Tokyo, Japan). Ar and N_2_ gases were used with a purity of 99.9999%, and the air was pre-purified using a HEPA filter.

Figure 1c shows an equivalent electrical circuit of the SDG. A spark discharge was initiated by applying a discharge voltage *U*_0_ to the electrodes through capacitor *C*, which was charged from a high voltage source. The capacitor was composed of 4700 pF low-inductance high-voltage pulse capacitors (KVI-3, ZVEK Progress, Ltd., Ukhta, Russia) connected in parallel. The total capacitance was *C* = 107 nF. The electrical circuit had some parasitic inductance and resistance, which were *L* = 1.13 μH and *R*_circuit_ = 110 mΩ, respectively. The discharge voltage *U*_0_ and the repetition rate of discharge *f* were kept constant by maintaining a constant breakdown gap between the electrodes *d*_gap_ and the capacitor charging current and were ~2.3 kV and 250 Hz, respectively. The breakdown gaps *d*_gap_ between the electrodes were 3.0 mm for Ar, and 0.7 mm for N_2_ and air, respectively. The spark discharge voltage *U*(*t*) was measured with a capacitive-resistive voltage divider connected in parallel with the capacitor, see Figure 1c. The signal from the voltage divider was given to an oscilloscope (DPO 4102B-L, Tektronix, Inc., Beaverton, OR, USA). The energy stored in the capacitor *E* was determined according to the following Equation (1):

(1) $$E=\frac{C\cdot U_{0}{}^{2}}{2},$$

where *C*—capacitance; *U*_0_—capacitor (discharge) voltage.

In experiments on the synthesis of Pt nanoparticles, the energy stored in the capacitor was ~283 mJ. The collection of synthesized nanoparticles for the preparation of nano-ink was carried out on an AFA-RMV-20 filter with a particle capture efficiency of more than 99.995%.

### 2.2. Preparation and Testing of Nano-Ink

In experiments on the preparation of platinum nano-ink, ethylene glycol (EG) with polyvinylpyrrolidone (PVP) was used as a medium for dispersing nanoparticles. The chemical reagents were of analytical purity without additional purification. Ultrasonic treatment with a specific power of about 3 W/cm^3^ for 90 min was used to deagglomerate particles in nano-ink. Moreover, the coarse fraction of particles was additionally removed from the nano-ink using sedimentation in the gravitational field within 24 h. The required values of surface tension and viscosity of nano-ink were achieved by changing the concentration of nanoparticles and binder. The prepared Pt nano-ink was tested using a commercial aerosol jet printer (AJ 15XE, Neotech AMT GmbH, Nuremberg, Germany) to form narrow (30 μm) and highly conductive lines on a ceramic alumina substrate. In these experiments, platinum nano-ink was sprayed with a pneumatic atomizer and deposited onto a moving substrate through a coaxial micro-nozzle with a diameter of 150 μm. The speed of substrate was 100 mm/min. The width of the printed line *w* was set by the aerosol (25 sccm) and sheath (20 sccm) flow rates through the coaxial micro-nozzle. The spreading of nano-ink was controlled by the substrate heating temperature *T*_s_ in the range from 25 to 150 °C. Then, printed lines were sintered in air atmosphere in the muffle furnace at the temperature range from 450 to 900 °C. The total sintering time was 120 min.

### 2.3. Characterization of Nanoparticles, Nano-Ink and Printed Lines

The morphology, size, and crystal structure of the synthesized nanoparticles were investigated by transmission electron microscope (TEM) (JEM-2100, JEOL Ltd., Tokyo, Japan). The elemental composition of nanoparticles was determined using energy dispersive X-ray spectroscopy (EDX) in a scanning electron microscope (SEM) (JSM-7001F, JEOL). The particle size distribution at the outlet from the SDG was measured with an aerosol spectrometer (SMPS 3936, TSI Inc., Shoreview, MN, USA) in real-time. X-ray photoelectron spectroscopy (XPS) was used to determine the chemical composition and oxidation state of nanoparticles by photoelectron spectrometer (Theta Probe, Thermo Scientific Inc., Waltham, MA USA). The mass production rate of the synthesis of nanoparticles *m* in various gases was estimated by the gravimetric method using an analytical balance (Secura 225D-1ORU, Sartorius Inc., Goettingen, Germany). The surface tension and viscosity of the nano-ink were analyzed by an optical tensiometer (DSA25S, Krüss GmbH, Hamburg, Germany) and a viscometer (SV-10, A&D Company, Limited, Tokyo, Japan), respectively. The resistivity of the sintered lines *ρ* was calculated using the following Equation (2):

(2) $$\rho =\frac{R\cdot S}{L},$$

where *R*—electrical resistance; *S*—cross-sectional area; *L*—length.

The electrical resistance of the line *R* was measured using a 4-point method using a multimeter (U1253B, Agilent Technologies Inc., Santa Clara, CA, USA) and a precision current source (SourceMeter 2401, Tektronix Inc., Beaverton, OR, USA). The cross-sectional area *S* and the length *L* of the line were measured using an optical 3D profilometer (S neox, Sensofar, Terrassa, Spain).

## 3. Results and Discussion

### 3.1. Results of Characterization of Synthesized Nanoparticles

**Figure 2:**
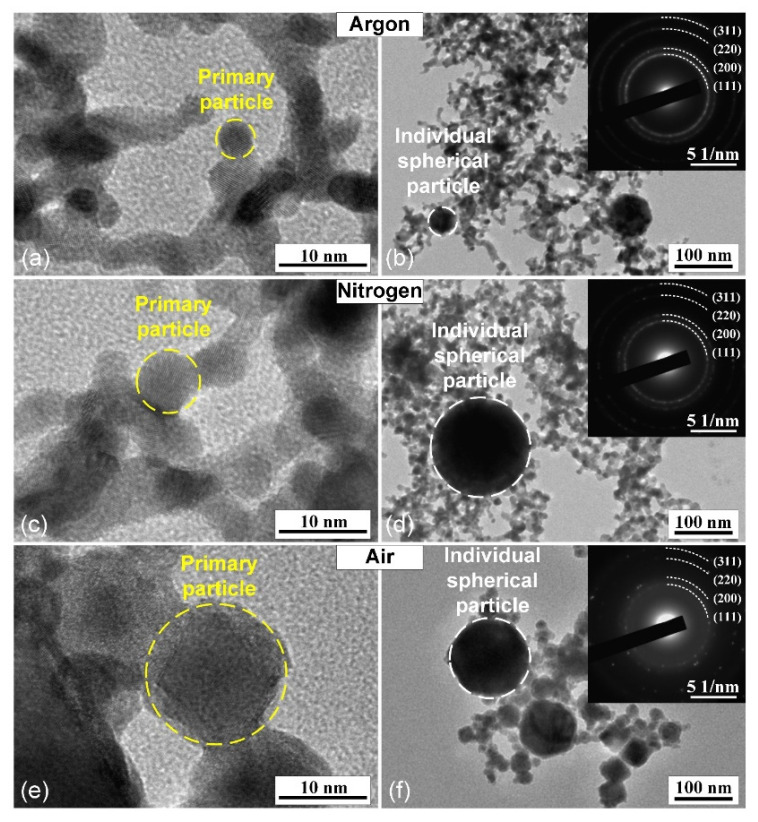
TEM images of aggregates, primary and individual spherical platinum nanoparticles (PtNPs) synthesized by SDG in various gases: (**a**,**b**) Ar, (**c**,**d**) N_2_, and (**e**,**f**) air. The insets show the corresponding electron diffraction patterns.

**Figure 3:**
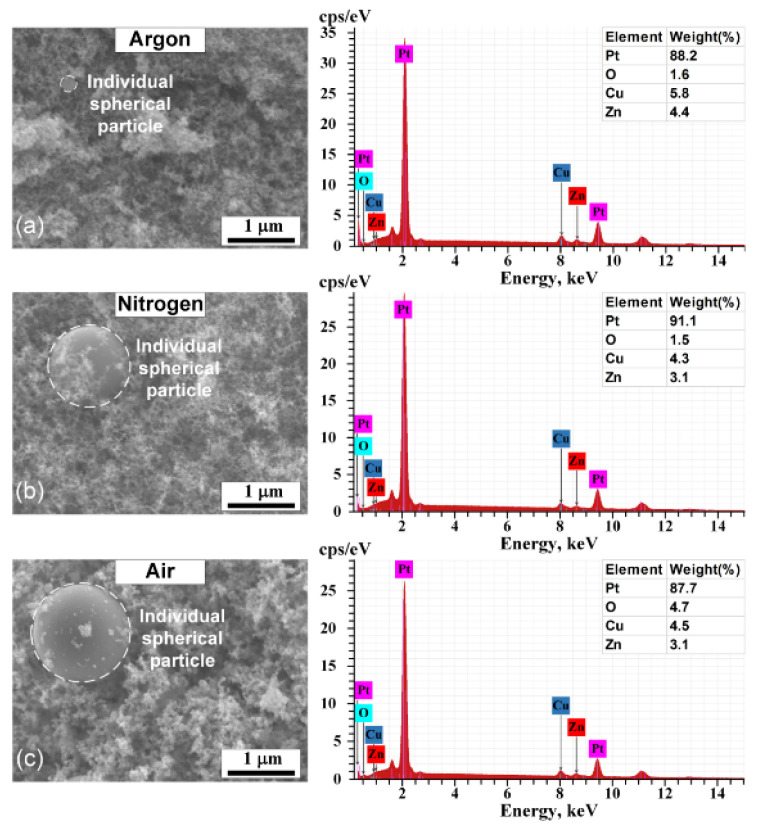
SEM images and EDX spectra of PtNPs synthesized by spark discharge under (**a**) Ar, (**b**) N_2_ and (**c**) air.

**Figure 4:**
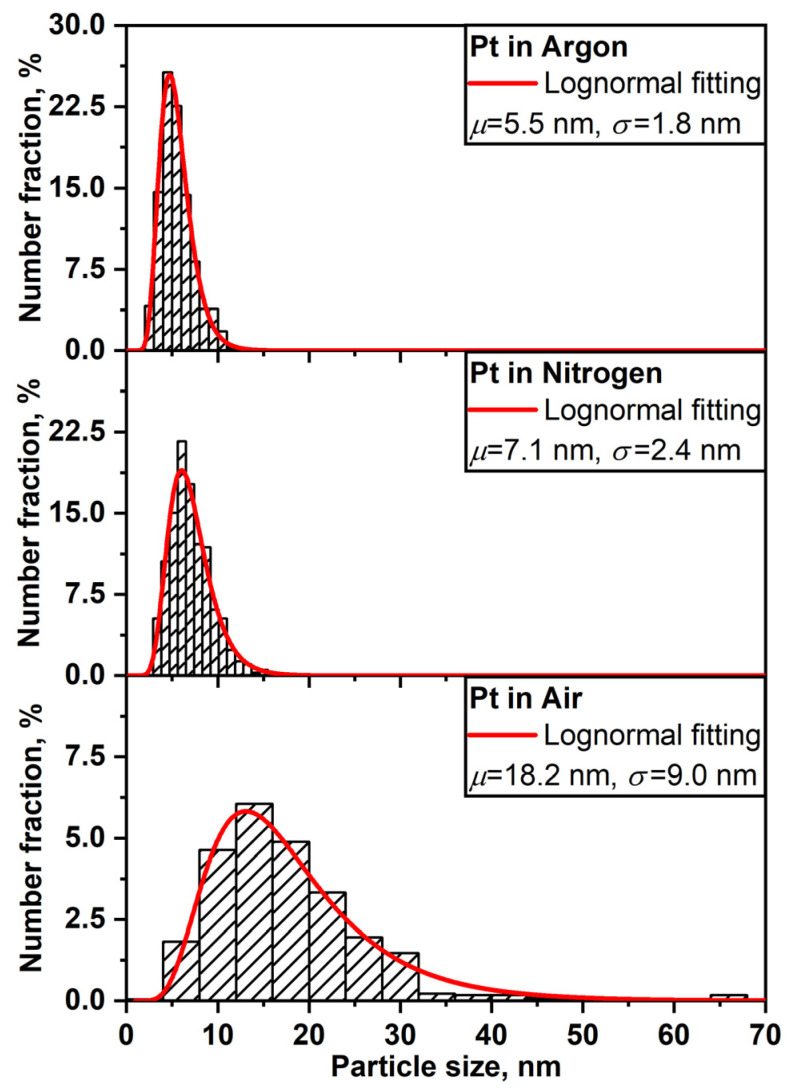
Histograms of the particle size distribution of primary Pt nanoparticles synthesized by spark discharge under Ar, N_2_, and air. Histograms are determined from TEM images and approximated by a lognormal function.

**Figure 5:**
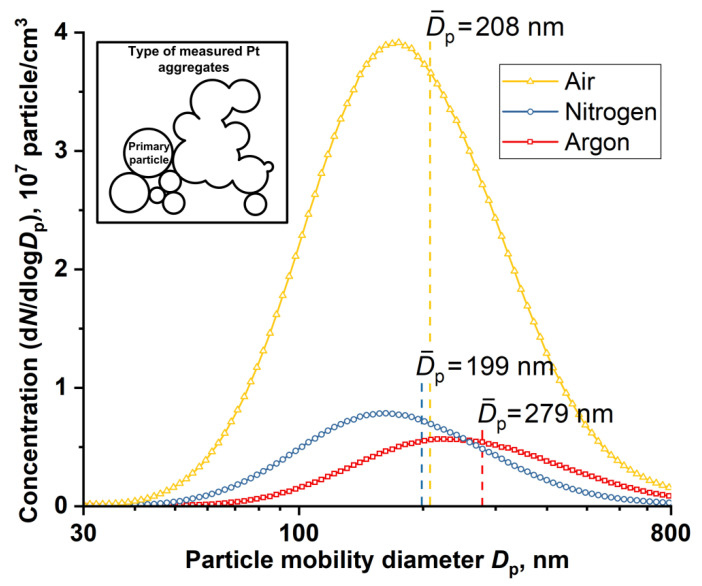
Particle size distributions of Pt aggregates synthesized by spark discharge under Ar, N_2_ and air. The measurements were performed using an aerosol spectrometer.

It is known that during the synthesis of nanoparticles by spark discharge, the parameters of the gas environment can affect the kinetics of the nucleation and growth of nanoparticles, which leads to the formation of nanoparticles with different morphology, size, and crystal structure [39,41]. Figure 2 and Figure 3 show TEM and SEM images of PtNPs synthesized by spark discharge under Ar, N_2_ and air. From the analysis of the image data, it was determined that, regardless of the carrier gas, the synthesized particles were primary nanoparticles with a round shape (~10 nm in diameter), grouped in aggregates with an average size of ~200 nm. The sizes of primary nanoparticles and aggregates were measured by TEM and aerosol spectrometer, respectively. The obtained platinum particles in the form of aggregates are typical for the synthesis of nanoparticles by spark discharge [52]. Additionally, it was determined that regardless of the carrier gas, the synthesized material also contains large individual spherical particles (up to 10–20 wt.%) with a diameter of 40 to 800 nm. These particles are observed in images from the transmission and scanning electron microscopes, see Figure 2 and Figure 3. Probably, large particles were formed as a result of splashing the molten material of the electrodes [52,53]. Figure 4 shows particle size distribution histograms for primary platinum nanoparticles, measured with a TEM. According to the results of TEM analysis, it was determined that the average sizes of primary PtNPs *μ* synthesized under Ar, N_2_, and air were 5.5 ± 1.8, 7.1 ± 2.4, and 18.2 ± 9.0 nm, respectively. At the same time, aggregates of primary PtNPs had an average size ${\bar{D}}_{p}$ equal to 199–279 nm, according to measurements of an aerosol spectrometer, see Figure 5. Figure 4 and Figure 5 show that the particle size distributions of primary and aggregates of primary PtNPs are well approximated by a log-normal function. From the analysis of electron diffraction patterns, it was found that the synthesized PtNPs contain a crystalline phase, regardless of the carrier gas. Thus, circular reflections corresponding to interplanar distances of 2.25, 1.94, 1.36 and 1.17 Å are present in the electron diffraction patterns, see Figure 2b,d,f). These reflections coincide with the interplanar distances of Pt with a cubic lattice of the space group Fm3m from the planes (111), (200), (220), and (311), respectively.

**Figure 6:**
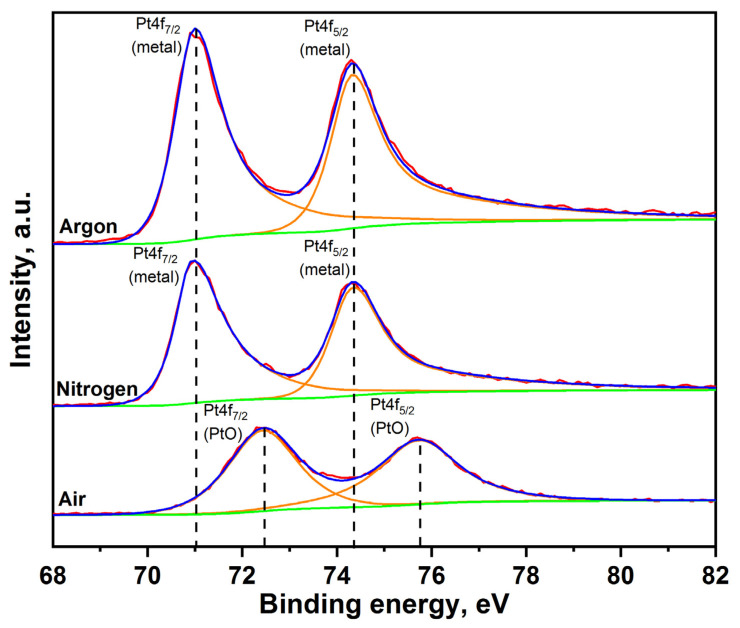
X-ray photoelectron spectra of PtNPs synthesized by spark discharge under Ar, N_2_ and air.

**Figure 7:**
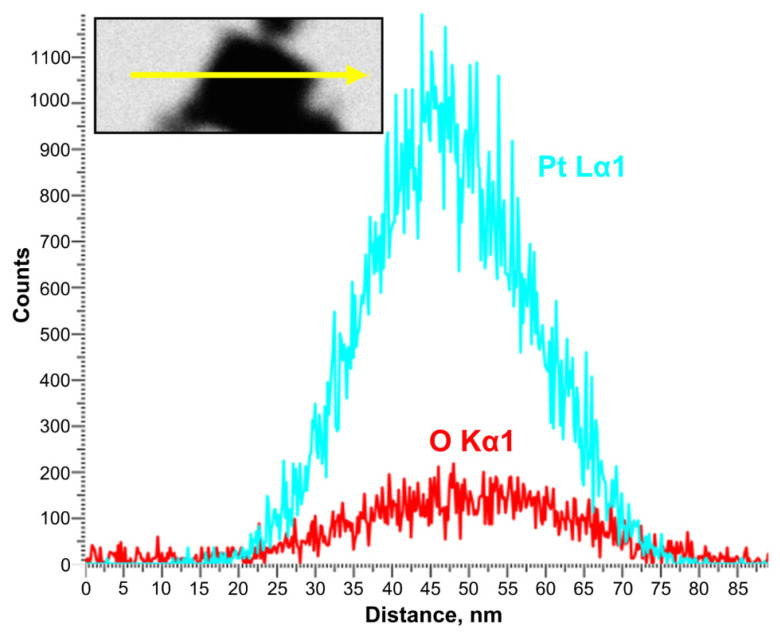
EDX profile of Pt nanoparticle synthesized by spark discharge under air. The inset shows the TEM image of the measured platinum nanoparticle and the corresponding scan line.

Despite the existing similarities in the parameters of PtNPs synthesized in various gases, key differences in their physical and chemical characteristics have also been identified. For example, in Figure 2 it can be seen that “air” nanoparticles have more complex rounded and rhombic shapes with a core-shell structure. At the same time, the average size of primary nanoparticles synthesized under air is 3 times larger than the size of nanoparticles produced under Ar and N_2_, see Figure 2. The elemental composition of synthesized PtNPs also differs according to the results of EDX, see Figure 3. So, the oxygen concentration in the samples of PtNPs synthesized in an air atmosphere (4.7 wt.%) is higher than in the nanoparticles synthesized under Ar (1.6 wt.%) and N_2_ (1.5 wt.%). Additionally, it was determined from the results of XPS that the samples synthesized in an air atmosphere contain platinum oxide PtO, see Figure 6. This is indicated by the presence of two characteristic peaks with binding energies equal to 72.4 eV and 75.7 eV [54,55]. At the same time, the samples synthesized under Ar and N_2_ are metallic platinum with zero-valence state and binding energies equal to 71.2 eV and 74.3 eV [56]. An EDX-TEM scan shows a uniform increase in the concentration of oxygen and platinum when moving from the edge to the center of the nanoparticle, see Figure 7. This result indirectly confirms the presence of an amorphous oxide layer on PtNPs synthesized in an air atmosphere.

### 3.2. Results of Measurements of Mass Production Rate and Spark Energy Calculations

According to the results of gravimetric measurements, it was established that the mass production rates of the synthesis of nanoparticles *m* under Ar, N_2_ and air were 53 ± 6, 366 ± 59, and 490 ± 36 mg/h, respectively. This difference in *m* values can be related to the energy dissipated in the spark gap *E*_spark_. Indeed, the mass production rate depends on the amount of evaporated material from the electrodes, and this, in turn, is related to the energy dissipated in the spark gap. Under the assumption that the spark discharge is considered as a plasma with a constant resistance *R*_spark_ [57] to determine *E*_spark_, the following Equation (3) can be used:

(3) $$E_{\mathrm{spark}}=\frac{CU_{0}^{2}}{2}\left(\frac{R_{\mathrm{spark}}}{R_{\mathrm{spark}}+R_{\mathrm{circuit}\;}}\right),$$

where *C*—capacitance; *U_0_*—capacitor (discharge) voltage; *R*_spark_ and *R*_circuit_—plasma resistance of the spark gap and resistance of electrical connections, respectively.

**Figure 8:**
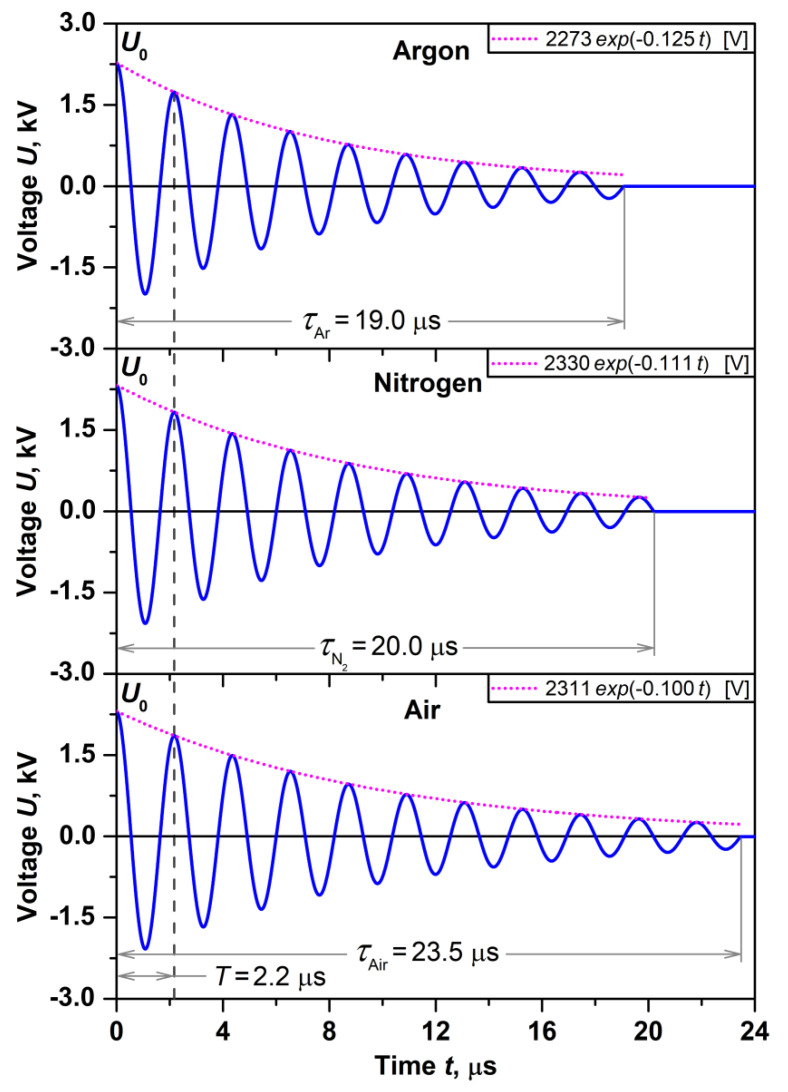
Oscillograms of voltages *U* (*t*) measured between platinum electrodes during a spark discharge under Ar, N_2_ and air.

Since in this approximation during the discharge *R*_spark_ is constant, the electric current in the spark gap and the circuit is the same. Consequently, the energy in the spark gap amounts to a certain fraction $\frac{R_{\mathrm{spark}}}{R_{\mathrm{spark}}+R_{\mathrm{circuit}\;}}$ from total energy $\frac{CU_{0}^{2}}{2}$, stored in the capacitor. Measuring *R*_spark_ is complicated, therefore, it is more practical to determine it by finding the damping coefficient *δ* from the approximation of the voltage oscillogram *U*(*t*) [58]. Figure 8 shows *U*(*t*) measured during a spark discharge between platinum electrodes under Ar, N_2_ and air.

Assuming that the electrical scheme is an RLC circuit, *R*_spark_ was calculated according to the following Equation (4):

(4) $$R_{\mathrm{spark}}=2\delta L-R_{\mathrm{circuit}},$$

where *δ*—damping coefficient; *L*—inductance of electrical connections.

The main parameters of the circuit: resistance *R*_circuit_, inductance of electrical connections *L*, and capacitance *C* were 110 mΩ, 1.13 μH, and 107 nF, respectively. The following equation was used as an approximating function for oscillograms (5):

(5) $$U\left(t\right)=U_{0}\;cos\left(\frac{2\pi t}{T}\right)exp\left(-\delta t\right),$$

where *t* and *T* are the time and period of voltage oscillations, respectively.

From the presented oscillograms it can be seen that the discharge in the air atmosphere has the longest duration of oscillations (*τ* = 23.5 μs)*,* see Figure 8. Long-term oscillations are the result of low values of *R*_spark_, and, as a consequence, are characterized by low values of energy fraction, dissipated in the spark gap $\frac{R_{spark}}{R_{\mathrm{spark}}+R_{\mathrm{circuit}\;}}$. The values of the total energy stored in the capacitor $\frac{CU_{0}^{2}}{2}$ in accordance with the measured discharge voltages *U*_0_ under argon (2273 V), nitrogen (2330 V), and air (2311 V) were 276, 290, and 286 mJ, respectively. Thus, the calculated *E*_spark_ values under Ar, N_2_ and air were 169, 163, and 147 mJ, respectively. In turn, low values of *E*_spark_ should be characterized by low values of *m*. However, according to the results of gravimetric measurements of *m*, an inverse relationship was obtained, see Table 1.

Thus, it can be assumed that the energy dissipated in the spark gap is not the main parameter that determines the mass production rate. Recently [52,59,60] it was reported that the mass production rate can be influenced by various gas parameters, such as heat capacity, thermal conductivity, and others. At the same time, similar to the presented results, other researchers also observed a higher mass production rate under air and N_2_ [53,61].

### 3.3. Results on Preparation and Printing with Nano-Ink

**Figure 9:**
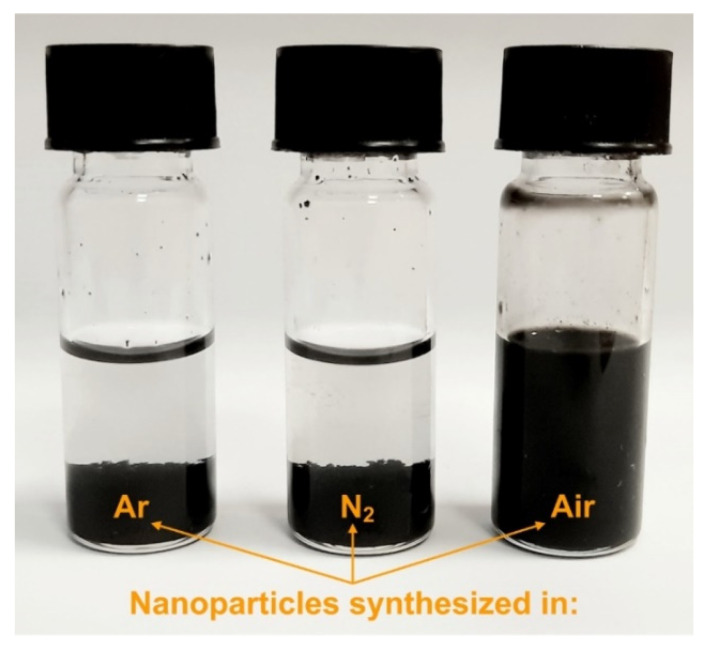
Photo of the prepared suspensions (nano-inks) with Pt nanoparticles (25 wt.%) dispersed in ethylene glycol (EG) with polyvinylpyrrolidone (PVP).

It is obvious that the found differences in the physical and chemical characteristics of nanoparticles can affect the possibility and technique of preparing nano-ink for aerosol jet printing (AJP). In this paper, nano-ink was prepared on the basis of synthesized PtNPs using identical solvent and a binder of ethylene glycol (EG) and polyvinylpyrrolidone (PVP), respectively. Platinum nanoparticles synthesized by spark discharge under Ar, N_2_ and air were used as a starting material for the preparation of nano-ink. It was found experimentally that stable Pt nano-ink was obtained only on the basis of nanoparticles synthesized under air atmosphere. Figure 9 shows a photo of suspensions (nano-inks) with various Pt nanoparticles dispersed in EG with PVP.

Figure 9 shows that metallic PtNPs synthesized under Ar and N_2_ turned out to be aggregatively and sedimentary unstable. These nanoparticles aggregated and precipitated as a result of their dispersion in EG solvent. On the other hand, oxidized nanoparticles synthesized in the air were efficiently dispersed in EG solvent and, as a consequence, formed stable platinum nano-ink [62,63], see Figure 9. This difference in the stabilization of nanoparticles is probably due to the fact that platinum metal nanoparticles have a lower affinity for the polar solvent used in comparison with oxidized nanoparticles. [64,65]. For this reason, other solvents and stabilization approaches must be used to disperse platinum metal nanoparticles synthesized under Ar and N_2_ [66].

In view of this, further experiments to optimize the composition of nano-ink for AJP were carried out only using oxidized PtNPs synthesized in an air atmosphere. Based on the results of optimization studies using nanoparticles synthesized under air, it was determined that the concentrations of nanoparticles and binder in nano-ink for the implementation of the AJP process should be 25 wt.% and 4 wt.%, respectively. In this case, the surface tension and viscosity of the optimized nano-ink were 43.9 mN/m and 11.4 cP, respectively. The optimized composition and parameters of Pt nano-ink based on nanoparticles synthesized by spark discharge under air are presented in Table 2.

**Figure 10:**
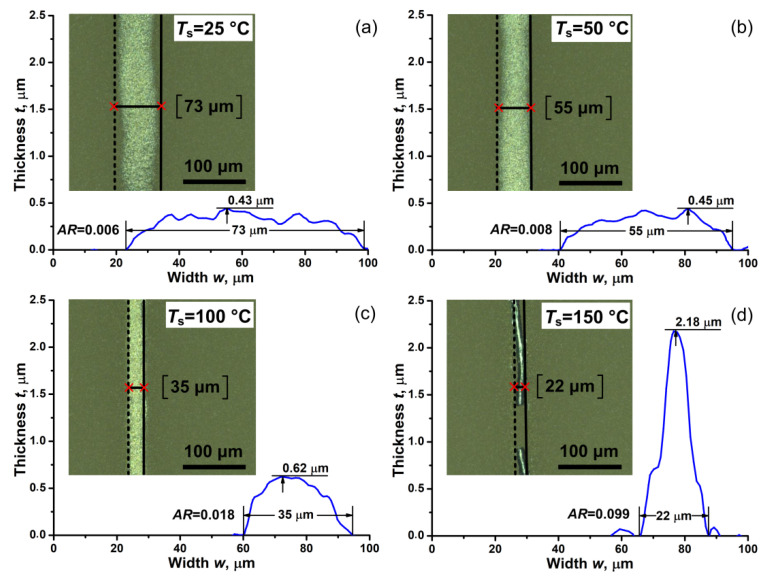
Profiles and corresponding optical images (on inserts) of platinum lines formed by AJP at various values of *T*_s_ equal to (**a**) 25 °C, (**b**) 50 °C, (**c**) 100 °C, and (**d**) 150 °C.

Experiments on the formation of narrow (about 30 μm) and conductive Pt lines were performed in order to optimize the parameters of nano-ink for AJP process [67,68]. Such lines are in demand as high-temperature and corrosion-resistant elements of various printed sensors [47,48,69]. The formation of Pt lines was carried out using the technology of AJP on a heat-resistant alumina substrate. The spreading and evaporation of nano-ink were controlled by heating the substrate temperature *T*_s_ in the range from 25 to 150 °C [70]. At the same time, the electrical resistance of the lines *R* was changed by controlling the number of printing layers in the range of 4–10 layers. In addition, in order to remove the solvent and form electrical contacts between the particles, they were thermally sintered in a muffle furnace. Sintering was carried out in an air at the temperatures range *T*_sint_ from 450 to 900 °C for 120 min. Figure 10 shows the 2D profiles and optical images of Pt lines formed by AJP onto a heated substrate at *T*_s_ from 25 °C to 150 °C. Figure 10 shows that the printed line width *w* decreases significantly from 73 to 22 μm with an increase in the heating of *T*_s_ from 25 to 150 °C, respectively. It is also seen that the aspect ratio *AR* (thickness/width) of the lines increases 16.5 times with an increase in the substrate temperature. This decrease in *w* and an increase in the aspect ratio is probably associated with an increase in the contact angle *θ* as a result of a change in the composition of the nano-ink due to the evaporation of the solvent during the deposition of the nano-ink on a heated substrate. Recently [71,72] it was found that the contact angle can vary over a wide range from 30° to >90° depending on *T*_s_ and thus largely determine the geometry of the lines being formed. Thus, from the analysis of the optical images of the lines, it was found that the optimal values of *T*_s_ during the printing process should be about 50–100 °C. In this temperature range, printed lines are formed with high *AR* values and without significant defects and breaks.

**Figure 11:**
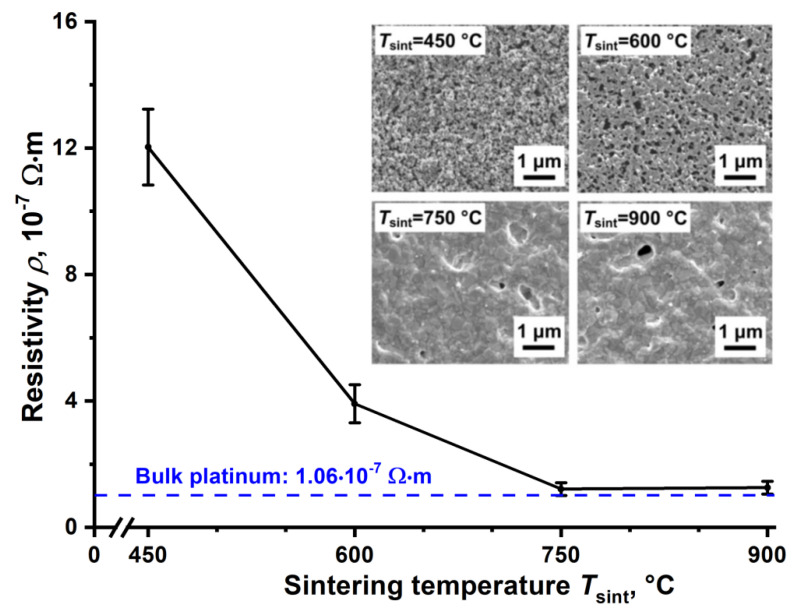
Dependence of the resistivity of platinum lines on *T*_sint_ and the corresponding SEM images of the surface of the sintered lines (inset).

Figure 11 shows the dependence of the resistivity of platinum lines on the sintering temperature *T*_sint_. The minimum value of *ρ* on an alumina substrate was (1.2 ± 0.1)·10^−7^ Ω∙m after sintering at 750 °C. The achieved value of *ρ* is about 1.1 times higher than the resistivity of bulk platinum 1.06·10^−7^ Ω∙m. In comparison with other studies [73,74,75,76], the developed platinum nano-ink shows lower values of resistivity after thermal sintering, see Table 3.

**Figure 12:**
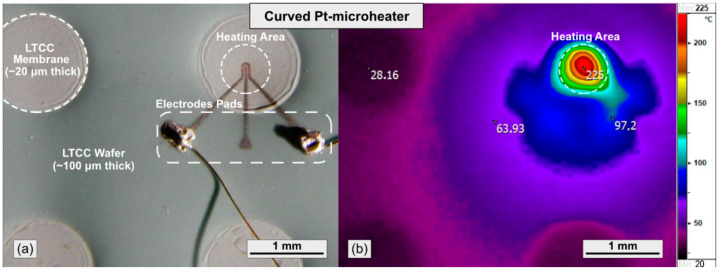
(**a**) Photo and (**b**) thermogram of curved Pt-microheater printed on a 20 μm thick low-temperature co-fired ceramic (LTCC) membrane.

This is probably achieved due to the low residual porosity and high chemical purity of the sintered material at *T*_sint_ > 600 °C. Indeed, the inset to Figure 11 shows that the Pt lines sintered at 750–900 °C have a high degree of sintering and low residual porosity. Additionally, it can be seen from comparative Table 3 that the developed nano-ink has a high concentration of particles (25 wt.%) in comparison with other studies [73,74,75,76], see Table 3. The use of concentrated nano-ink is an additional advantage since it allows reducing the time for printing lines with a given resistance. Thus, the low resistivity and high concentration of Pt nano-ink confirm the reasonableness of the developed method for preparing nano-ink using the synthesis of nanoparticles by spark discharge. Prepared platinum nano-ink can be promising for printing current-carrying contacts, elements of gas, temperature and biological sensors. In order to demonstrate the possibility of using nano-ink in addition to single lines, we formed curved Pt-microheaters, see Figure 12.

Figure 12a shows a photo of curved Pt-microheater printed on a 20-μm-thick low-temperature co-fired ceramic (LTTC) membrane and sintered at 750 °C in a muffle furnace. The stencil of Pt-microheater was developed and presented in previous work [77]. This microheater had a curved shape and a heating line width of about 30 μm in the narrow part. The electrodes pads were applied to the wide lateral parts of the microheater to fed by power supply SourceMeter 2401 (Tektronix, Inc., Beaverton, OR, USA). Figure 12b shows a thermogram of an operating microheater, measured by Thermograph IRTIS-2000 (IRTIS, Ltd., Moscow, Russia). The heating area of the microheater has a maximum temperature of 225 °C at a power consumption of 50 mW (2.1 V). However, the operating temperature of the microheater can be easily increased up to 400–500 °C by increasing the power consumption, which is required for its use in resistive sensors [75,78]. Similar microstructures can be used as low-cost printed sensors (temperature, gas and others) for various electronic and non-electronic devices with a facile manufacturing process.

## 4. Conclusions

The synthesis of PtNPs by spark discharge can be used to prepare highly conductive nano-ink for printed electronics. Synthesis of nanoparticles by spark discharge is a cost-effective, environmentally friendly and scalable method that does not require chemically hazardous precursors. Depending on the type of carrier gas (Ar, N_2_ and air) and the spark discharge parameters, the mass production rate of PtNPs can be equal to 53–490 mg/h. However, the mass production rate depends more on the type of carrier gas rather than on the energy dissipated in the spark gap. The synthesized materials, regardless of the carrier gas, are near-spherical primary nanoparticles (10 nm in diameter) grouped into submicron aggregates. Furthermore, crystal metallic Pt nanoparticles can be generally synthesized under Ar and N_2_, whereas amorphous PtO nanoparticles can be formed under air atmosphere. Sedimentation and aggregation stability of platinum oxide nanoparticles in ethylene glycol has been observed. Thus, PtNPs synthesized under air are suitable for preparing platinum nano-ink. The prepared Pt nano-ink is tested to form narrow (up to 30 μm) and highly conductive platinum lines using aerosol jet printing. The resistivity of the sintered Pt lines is (1.2 ± 0.1)·10^−7^ Ω·m, which is about 1.1 times higher than that of bulk Pt. Thus, the prepared platinum nano-ink can be promising for printing corrosion-resistant current-carrying contacts, elements of gas, temperature and biological sensors.

## Figures and Tables

**Table 1:** Mass production rate, time and energy parameters of the spark discharge depending on the carrier gas.

Carrier Gas	Mass Production Rate *m* [mg/h]	Damping Coefficient *δ* [(µs)^−1^]	Plasma Resistance *R*_spark_ [mΩ]	Energy Dissipated in the Spark Gap *E*_spark_ [mJ]	Energy Stored in the Capacitor $\frac{CU_{0}^{2}}{2}\;[\mathrm{mJ}]$
Argon	53 ± 6	0.125	173	169	276
Nitrogen	366 ± 59	0.111	141	163	290
Air	490 ± 36	0.100	116	147	286

**Table 2:** Platinum nano-ink parameters optimized for aerosol jet printing (AJP) process.

**Composition**	
Ethylene glycol (EG), wt.%	71
Polyvinylpyrrolidone (PVP), wt.%	4
Pt nanoparticles, wt.%	25
**Parameters**	
Surface tension, mN/m	43.9
Viscosity, cP	11.4

**Table 3:** Comparison of parameters of platinum nano-ink.

Reference	Particle Size, nm	Concentration, wt.%	Substrate Material	Sintering Temperature *T*_sint_, °C	Resistivity *ρ*, 10^−7^ Ω·m
Vasiliev et al., 2018 [73]	3–8	10–20	Alumina	800	30
Pandhi et al., 2020 [74]	5–8	20	Kapton	425	13
Kassem et al., 2019 [75]	6	5	Polyimide	350	3.5
Kirbus et al., 2018 [76]	3	10	Silica	300	3.0
This work	18	25	Alumina	750	1.2

## Data Availability

The data presented in this study are available on request from the corresponding author.

## References

[1] Kim S. (2020). Inkjet-Printed Electronics on Paper for RF Identification (RFID) and Sensing. Electronics.

[2] Chen Y.-D., Nagarajan V., Rosen D.W., Yu W., Huang S.Y. (2020). Aerosol jet printing on paper substrate with conductive silver nano material. J. Manuf. Process..

[3] Chen J., Mishra S., Vaca D., Kumar N., Yeo W.-H., Sitaraman S.K., Kumar S. (2020). Thin dielectric-layer-enabled low-voltage operation of fully printed flexible carbon nanotube thin-film transistors. Nanotechnology.

[4] Ye H., Kwon H.-J., Tang X., Lee D.Y., Nam S., Kim S.H. (2020). Direct Patterned Zinc-Tin-Oxide for Solution-Processed Thin-Film Transistors and Complementary Inverter through Electrohydrodynamic Jet Printing. Nanomaterials.

[5] Wang C.-J., You H.-C., Ou J.-H., Chu Y.-Y., Ko F.-H. (2020). Ultraviolet Photodetecting and Plasmon-to-Electric Conversion of Controlled Inkjet-Printing Thin-Film Transistors. Nanotechnology.

[6] Antonova I.V., Shavelkina M.B., Ivanov A.I., Soots R.A., Ivanov P.P., Bocharov A.N. (2020). Graphene Flakes for Electronic Applications: DC Plasma Jet-Assisted Synthesis. Nanotechnology.

[7] Borghetti M., Serpelloni M., Sardini E. (2019). Printed Strain Gauge on 3D and Low-Melting Point Plastic Surface by Aerosol Jet Printing and Photonic Curing. Sensors.

[8] Keller P., Kawasaki H. (2021). Conductive leaf vein networks produced via Ag nanoparticle self-assembly for potential applications of flexible sensor. Mater. Lett..

[9] Deiner L.J., Jenkins T., Powell A., Howell T., Rottmayer M. (2019). High Capacity Rate Capable Aerosol Jet Printed Li-Ion Battery Cathode. Adv. Eng. Mater..

[10] Kim K.-W., Kim Y.M., Li X., Ha T., Kim S.H., Moon H.C., Lee S.W. (2020). Various Coating Methodologies of WO_3_ According to the Purpose for Electrochromic Devices. Nanotechnology.

[11] Bag S., Deneault J.R., Durstock M.F. (2017). Aerosol-Jet-Assisted Thin-Film Growth of CH_3_NH_3_PbI_3_ Perovskites-A Means to Achieve High Quality, Defect-Free Films for Efficient Solar Cells. Adv. Energy Mater..

[12] Eggers H., Schackmar F., Abzieher T., Sun Q., Lemmer U., Vaynzof Y., Richards B.S., Hernandez-Sosa G., Paetzold U.W. (2020). Inkjet-Printed Micrometer-Thick Perovskite Solar Cells with Large Columnar Grains. Adv. Energy Mater..

[13] Beedasy V., Smith P.J. (2020). Printed Electronics as Prepared by Inkjet Printing. Materials.

[14] Kwon J.-S., Lee D.J., Oh J.H. (2018). Formation and Characterization of Inkjet-Printed Nanosilver Lines on Plasma-Treated Glass Substrates. Appl. Sci..

[15] Pandhi T., Chandnani A., Subbaraman H., Estrada D. (2020). A Review of Inkjet Printed Graphene and Carbon Nanotubes Based Gas Sensors. Sensors.

[16] Yang P., Fan H.J. (2020). Inkjet and Extrusion Printing for Electrochemical Energy Storage: A Minireview. Adv. Mater. Technol..

[17] Wilkinson N.J., Smith M.A.A., Kay R.W., Harris R.A. (2019). A review of aerosol jet printing—A non-traditional hybrid process for micro-manufacturing. Int. J. Adv. Manuf. Technol..

[18] Mensing J.P., Lomas T., Tuantranont A. (2020). 2D and 3D printing for graphene based supercapacitors and batteries: A review. Sustain. Mater. Technol..

[19] Ćatić N., Wells L., Al Nahas K., Smith M., Jing Q., Keyser U.F., Cama J., Kar-Narayan S. (2020). Aerosol-jet printing facilitates the rapid prototyping of microfluidic devices with versatile geometries and precise channel functionalization. Appl. Mater. Today.

[20] Arsenov P.V., Efimov A.A., Ivanov V.V. (2020). Comparison of Thermal and Electrical Sintering of Aerosol Silver Nanoparticles in Process of Aerosol Jet Printing. Key Eng. Mater..

[21] Wang L., Wu Z., Cao C. (2019). Technologies and Fabrication of Intelligent Packaging for Perishable Products. Appl. Sci..

[22] Janczak D., Zych M., Raczyński T., Dybowska-Sarapuk L., Pepłowski A., Krzeminski J., Sosna-Głębska A., Znajdek K., Sibinski M., Jakubowska M. (2019). Stretchable and Washable Electroluminescent Display Screen-Printed on Textile. Nanotechnology.

[23] Andrews J.B., Cao C., Brooke M.A., Franklin A.D. (2017). Noninvasive Material Thickness Detection by Aerosol Jet Printed Sensors Enhanced Through Metallic Carbon Nanotube Ink. IEEE Sensors J..

[24] Nitta K., Ishizumi K., Shimizu Y., Terashima K., Ito T. (2021). One-step gold line fabrication from particle-free inorganic salt-based ink via atmospheric pressure nonequilibrium plasma-assisted inkjet printing. Mater. Chem. Phys..

[25] Wang Y., Wang H., Liu F., Wu X., Xu J., Cui H., Wu Y., Xue R., Tian C., Zheng B. (2020). Flexible printed circuit board based on graphene/polyimide composites with excellent thermal conductivity and sandwich structure. Compos. Part A Appl. Sci. Manuf..

[26] Mohammed M.G., Kramer R. (2017). All-Printed Flexible and Stretchable Electronics. Adv. Mater..

[27] Huang Q., Al-Milaji K.N., Zhao H. (2018). Inkjet Printing of Silver Nanowires for Stretchable Heaters. ACS Appl. Nano Mater..

[28] Huang Q., Zhu Y. (2019). Printing Conductive Nanomaterials for Flexible and Stretchable Electronics: A Review of Materials, Processes, and Applications. Adv. Mater. Technol..

[29] Fernandes D.F., Majidi C., Tavakoli M. (2019). Digitally printed stretchable electronics: A review. J. Mater. Chem. C.

[30] Fernandes I.J., Aroche A.F., Schuck A., Lamberty P., Peter C.R., Hasenkamp W., Rocha T.L.A.C. (2020). Silver nanoparticle conductive inks: Synthesis, characterization, and fabrication of inkjet-printed flexible electrodes. Sci. Rep..

[31] Cho C.H., Shin I.K., Kim K.Y., Choi Y.J. (2019). Enhancing adhesion properties between binder-free copper nanoink and flexible substrate using chemically generated interlocking structure. Appl. Surf. Sci..

[32] So M.-H., Ho C.-M., Chen R., Che C.-M. (2010). Hydrothermal Synthesis of Platinum-Group-Metal Nanoparticles by Using HEPES as a Reductant and Stabilizer. Chem. Asian J..

[33] Zhuo L., Liu W., Zhao Z., Yin E., Li C., Zhou L., Zhang Q., Feng Y., Lin S. (2020). Cost-effective silver nano-ink for inkjet printing in application of flexible electronic devices. Chem. Phys. Lett..

[34] Jeyaraj M., Gurunathan S., Qasim M., Kang M.-H., Kim J.-H. (2019). A Comprehensive Review on the Synthesis, Characterization, and Biomedical Application of Platinum Nanoparticles. Nanotechnology.

[35] Feng J., Chen D., Sediq A.S., Romeijn S., Tichelaar F.D., Jiskoot W., Yang J., Koper M.T.M. (2018). Cathodic Corrosion of a Bulk Wire to Nonaggregated Functional Nanocrystals and Nanoalloys. ACS Appl. Mater. Interfaces.

[36] Gehr P., Zellner R. (2019). Biological Responses to Nanoscale Particles: Molecular and Cellular Aspects and Methodological Approaches. NanoScience and Technology.

[37] Gu Y., Wu A., Federici J.F. (2017). Comparison of thermal decomposition and chemical reduction of particle-free silver ink for inkjet printing. Thin Solid Films.

[38] Messing M.E. (2016). The Advantages of Spark Discharge Generation for Manufacturing of Nanoparticles with Tailored Properties. J. Green Eng..

[39] Mylnikov D., Efimov A.A., Ivanov V. (2019). Measuring and optimization of energy transfer to the interelectrode gaps during the synthesis of nanoparticles in a spark discharge. Aerosol Sci. Technol..

[40] Ivanov V., Efimov A.A., Myl’Nikov D.A., Lizunova A.A. (2018). Synthesis of Nanoparticles in a Pulsed-Periodic Gas Discharge and Their Potential Applications. Russ. J. Phys. Chem. A.

[41] Krasnikov D.V., Zabelich B.Y., Iakovlev V.Y., Tsapenko A.P., Romanov S.A., Alekseeva A.A., Grebenko A.K., Nasibulin A.G. (2019). A spark discharge generator for scalable aerosol CVD synthesis of single-walled carbon nanotubes with tailored characteristics. Chem. Eng. J..

[42] Huang H., Wei Y., Yang Y., Yan M., He H., Jiang Q., Yang X., Zhu J. (2021). Controllable synthesis of grain boundary-enriched Pt nanoworms decorated on graphitic carbon nanosheets for ultrahigh methanol oxidation catalytic activity. J. Energy Chem..

[43] Ourari A., Zerdoumi R., Rosas J.M., Morallón E. (2019). Synthesis and Catalytic Properties of Modified Electrodes by Pulsed Electrodeposition of Pt/PANI Nanocomposite. Materials.

[44] Bettelli M., Amadè N.S., Zanettini S., Nasi L., Villani M., Abbene L., Principato F., Santi A., Pavesi M., Zappettini A. (2020). Improved electroless platinum contacts on CdZnTe X- and γ-rays detectors. Sci. Rep..

[45] Guo C.Y., Wan C.H., He W.Q., Zhao M.K., Yan Z.R., Xing Y.W., Wang X., Tang P., Liu Y.Z., Zhang S. (2020). A nonlocal spin Hall magnetoresistance in a platinum layer deposited on a magnon junction. Nat. Electron..

[46] Wang P., Tian X., Yan M., Yang B., Hua Z. (2021). A low temperature catalytic-type combustible gas sensor based on Pt supported zeolite catalyst films. J. Mater. Sci..

[47] Vasiliev A.A., Varfolomeev A.E., Volkov I.A., Simonenko N.P., Arsenov P.V., Vlasov I.S., Ivanov V.V., Pislyakov A.V., Lagutin A.S., Jahatspanian I. (2018). Reducing Humidity Response of Gas Sensors for Medical Applications: Use of Spark Discharge Synthesis of Metal Oxide Nanoparticles. Sensors.

[48] Chen J., Zhang J., Wang M., Li Y. (2014). High-temperature hydrogen sensor based on platinum nanoparticle-decorated SiC nanowire device. Sensors Actuators B Chem..

[49] Liu Z., Tian B., Fan X., Liu J., Zhang Z., Luo Y., Zhao L., Lin Q., Han F., Jiang Z. (2020). A temperature sensor based on flexible substrate with ultra-high sensitivity for low temperature measurement. Sensors Actuators A Phys..

[50] Cruz A.G., Haq I., Cowen T., Di Masi S., Trivedi S., Alanazi K., Piletska E., Mujahid A., Piletsky S.A. (2020). Design and fabrication of a smart sensor using in silico epitope mapping and electro-responsive imprinted polymer nanoparticles for determination of insulin levels in human plasma. Biosens. Bioelectron..

[51] De Oliveira G.C.M., Carvalho J.H.D.S., Brazaca L.C., Vieira N.C.S., Janegitz B.C. (2020). Flexible platinum electrodes as electrochemical sensor and immunosensor for Parkinson’s disease biomarkers. Biosens. Bioelectron..

[52] Němec T., Šonský J., Gruber J., De Prado E., Kupčík J., Klementova M. (2020). Platinum and platinum oxide nanoparticles generated by unipolar spark discharge. J. Aerosol Sci..

[53] Tabrizi N.S., Ullmann M., Vons V.A., Lafont U., Schmidt-Ott A. (2009). Generation of nanoparticles by spark discharge. J. Nanoparticle Res..

[54] Ono L.K., Yuan B., Heinrich H., Cuenya B.R. (2010). Formation and Thermal Stability of Platinum Oxides on Size-Selected Platinum Nanoparticles: Support Effects. J. Phys. Chem. C.

[55] Pootawang P., Saito N., Takai O., Lee S.-Y. (2012). Synthesis and characteristics of Ag/Pt bimetallic nanocomposites by arc-discharge solution plasma processing. Nanotechnology.

[56] Nichols W.T., Sasaki T., Koshizaki N. (2006). Laser ablation of a platinum target in water. III. Laser-induced reactions. J. Appl. Phys..

[57] Kohut A., Villy L., Ajtai T., Geretovszky Z., Galbács G. (2018). The effect of circuit resistance on the particle output of a spark discharge nanoparticle generator. J. Aerosol Sci..

[58] Palomares J.M., Kohut A., Galbács G., Engeln R., Geretovszky Z. (2015). A time-resolved imaging and electrical study on a high current atmospheric pressure spark discharge. J. Appl. Phys..

[59] Stein M., Kiesler D., Kruis F.E. (2013). Effect of carrier gas composition on transferred arc metal nanoparticle synthesis. J. Nanoparticle Res..

[60] Domaschke M., Schmidt M., Peukert W. (2018). A model for the particle mass yield in the aerosol synthesis of ultrafine monometallic nanoparticles by spark ablation. J. Aerosol Sci..

[61] Final Report Summary—BUONAPART-E (Better Upscaling and Optimization of Nanoparticle and Nanostructure Production by Means of Electrical Discharges). Report Summary. BUONAPART-E|FP7|CORDIS. European Commission. https://cordis.europa.eu/project/id/280765/reporting.

[62] Quinson J., Kacenauskaite L., Bucher J., Simonsen S.B., Kuhn L.T., Oezaslan M., Morsbach E., Arenz M. (2019). Controlled Synthesis of Surfactant-Free Water-Dispersible Colloidal Platinum Nanoparticles by the Co4Cat Process. ChemSusChem.

[63] Niederberger M., Pinna N. (2009). Solvent-Controlled Synthesis. Metal Oxide Nanoparticles in Organic Solvents: Synthesis, Formation, Assembly and Application.

[64] Iriarte-Mesa C., López Y.C., Matos-Peralta Y., De La Vega-Hernández K., Antuch M. (2020). Gold, Silver and Iron Oxide Nanoparticles: Synthesis and Bionanoconjugation Strategies Aimed at Electrochemical Applications. Top. Curr. Chem..

[65] Rossi L.M., Fiorio J.L., Garcia M., Ferraz C.P. (2018). The role and fate of capping ligands in colloidally prepared metal nanoparticle catalysts. Dalton Trans..

[66] Iida K., Sasabe T., Sakai K., Uemura S., Shinohara K., Hirai S. (2020). Effects of Solvent Composition on Viscosity and Dispersion Structure of PEFC Catalyst Ink. ECS Trans..

[67] Efimov A., Potapov G.N., Nisan A.V., Ivanov V.V. (2017). Controlled focusing of silver nanoparticles beam to form the microstructures on substrates. Results Phys..

[68] Arsenov P.V., Efimov A.A., Ivanov V.V. (2018). Effect of Methods of Changing in Focusing Ratio on Line Geometry in Aerosol Jet Printing. Key Eng. Mater..

[69] Sun L., Wang B., Wang Y. (2020). High-Temperature Gas Sensor Based on Novel Pt Single Atoms@SnO_2_ Nanorods@SiC Nanosheets Multi-heterojunctions. ACS Appl. Mater. Interfaces.

[70] Efimov A.A., Minkov K.N., Arsenov P.V., Protas N.V., Ivanov V.V. (2018). Investigation of sintering of silver lines on a heated plastic substrate in the dry aerosol jet printing. J. Phys. Conf. Ser..

[71] Efimov A., Arsenov P.V., Kornyushin D., Lizunova A.A., Volkov I.A., Ivanov V. (2020). Aerosol Jet Printing of Silver Lines with A High Aspect Ratio on A Heated Silicon Substrate. Materials.

[72] Kahng S.-J., Cerwyn C., Dincau B.M., Kim J.-H., Novosselov I.V., Anantram M.P., Chung J.-H. (2018). Nanoink bridge-induced capillary pen printing for chemical sensors. Nanotechnology.

[73] Vasiliev A., Kim V.P., Tkachev S.V., Kornilov D.Y., Gubin S.P., Vlasov I.S., Jahatspanian I., Sizov A.S. (2018). Platinum Based Material for Additive Technology of Gas Sensors. Proceedings.

[74] Pandhi T., Cornwell C., Fujimoto K., Barnes P., Cox J., Xiong H., Davis P.H., Subbaraman H., Koehne J.E., Estrada D. (2020). Fully inkjet-printed multilayered graphene-based flexible electrodes for repeatable electrochemical response. RSC Adv..

[75] Kassem O., Saadaoui M., Rieu M., Viricelle J.-P. (2019). A novel approach to a fully inkjet printed SnO_2_-based gas sensor on a flexible foil. J. Mater. Chem. C.

[76] Kirbus B., Brachmann E., Hengst C., Menzel S.B. (2018). Additive manufacturing of 96 MHz surface acoustic wave devices by means of superfine inkjet printing. Smart Mater. Struct..

[77] Arsenov P.V., Vlasov I.S., A Efimov A., Minkov K.N., Ivanov V.V. (2019). Aerosol Jet Printing of Platinum Microheaters for the Application in Gas Sensors. IOP Conf. Ser. Mater. Sci. Eng..

[78] Hermawan A., Asakura Y., Inada M., Yin S. (2020). A facile method for preparation of uniformly decorated-spherical SnO_2_ by CuO nanoparticles for highly responsive toluene detection at high temperature. J. Mater. Sci. Technol..

